# Rapid Detection and Typing of *Actinobacillus pleuropneumoniae* Serovars Directly From Clinical Samples: Combining FTA^®^ Card Technology With Multiplex PCR

**DOI:** 10.3389/fvets.2021.728660

**Published:** 2021-08-10

**Authors:** Oliver W. Stringer, Janine T. Bossé, Sonia Lacouture, Marcelo Gottschalk, László Fodor, Øystein Angen, Eduardo Velazquez, Paul Penny, Liancheng Lei, Paul R. Langford, Yanwen Li

**Affiliations:** ^1^Section of Paediatric Infectious Disease, Department of Infectious Disease, Imperial College London, London, United Kingdom; ^2^Groupe de Recherche sur les Maladies Infectieuses en Production Animale, Faculty of Veterinary Medicine, University of Montreal, Montreal, QC, Canada; ^3^Department of Microbiology and Infectious Diseases, University of Veterinary Medicine, Budapest, Hungary; ^4^Department of Microbiology and Infection Control, Statens Serum Institut, Copenhagen, Denmark; ^5^Ceva Animal Health Ltd., Amersham, United Kingdom; ^6^College of Veterinary Medicine, Jilin University, Changchun, China

**Keywords:** FTA card, DNA, *Actinobacillus pleuropeumoniae*, detection, diagnostics, multiplex-PCR

## Abstract

*Actinobacillus pleuropneumoniae* (APP), the causative agent of porcine pleuropneumonia, is highly contagious and responsible for high morbidity, mortality, and economic losses in the swine industry worldwide, but quick serotyping and diagnosis are still not widely available. In this study, we sought to validate the use of Whatman FTA^®^ cards for collection and processing of *A. pleuropneumoniae* isolates, or porcine lung tissue samples, for direct use in diagnostic multiplex PCRs. We have optimized the processing of 3-mm discs punched from FTA^®^ cards loaded with cultured *A. pleuropneumoniae*, or imprinted on lesioned regions of lung tissue, with only three distilled water washes before addition into our APP-multiplex PCR (mPCR) assay for rapid, low-cost identification and serotyping. DNA captured on FTA^®^ cards generated the same diagnostic PCR results as DNA extracted using commercial kits for 85 *A. pleuropneumoniae* clinical isolate cultures and 22 lung samples. Additionally, bacterial DNA bound to FTA^®^ cards was detectable by PCR after 6 months of storage at 37°C. This study provides simple, efficient, rapid, and practical sample processing for detection and molecular serotyping of *A. pleuropneumoniae*.

## Introduction

*Actinobacillus pleuropneumoniae* (APP) is an encapsulated Gram-negative bacterium and one of the most important respiratory pathogens affecting global swine production (1). Herds infected with *A. pleuropneumoniae* incur significant economic losses from increased mortality, reduced growth rates, high treatment costs, and/or eradication regimes (2, 3). Strategies to contain infection include good husbandry, high biosecurity measures, antimicrobial treatment, and vaccination (4–6). Isolates of *A. pleuropneumoniae* can be differentiated into serovars, based on differences in capsular polysaccharide antigens encoded by unique capsule synthesis (*cps*) genes (1, 7). Although all are capable of causing pleuropneumonia, some serovars are considered more pathogenic than others (8). Furthermore, regional differences in the distribution and prevalence of specific serovars can change over time, and since 2015, the number of known serovars of *A. pleuropneumoniae* has increased from 16 to 19, with the identification of novel *cps* loci in previously non-typable isolates (9–12). In the UK, serovar 8 has accounted for >70% of clinical isolates for more than a decade (13, 14), with serovars 2, 6, 7, and 12 also occasionally isolated. Whereas, in a survey of swine respiratory disease in northern Portugal, *A. pleuropneumoniae* was isolated in 26% of lesioned lungs but the serovars were not determined (15).

*A. pleuropneumoniae* serotyping is important for implementation and monitoring of vaccination programs, as the current vaccines (bacterins) provides little heterologous protection and the efficacy depends on correct coverage of serovar(s) circulating in the region at a given time. Recently, we validated two multiplex PCRs (APP-mPCR1 and APP-mPCR2) for detection and molecular typing of all 19 *A. pleuropneumoniae* serovars using DNA extracted from cultured bacteria (7, 12).

Direct detection of pathogenic bacteria from clinical tissue samples, or other culture-free methods, would greatly facilitate and expedite diagnostics. Collection and transportation of samples containing infectious agents from abattoirs or pig units to diagnostic laboratories pose potential risks to veterinary biosecurity. Therefore, strict legal requirements are placed on the transportation of biological materials, especially when shipping internationally, necessitating specialized couriers, which can be expensive and impractical in remote areas. Additional safety concerns arise in laboratories where personnel handle and process clinical samples in order to carry out diagnostic investigations. Thus, selecting a suitable medium for the sampling and inactivation of pathogens would be critical to provide risk-free transportation of infectious materials for diagnostic purposes.

Whatman FTA^®^ cards are a specialist form of filter paper, containing a chemical coating that lyses cells, denatures proteins, and binds nucleic acids, preventing their degradation by oxidation or UV radiation. FTA^®^ cards are commonly used to send clinical samples for molecular diagnostic purposes, as the inactivated pathogens are safe for transportation *via* standard postal routes, and the entrapped nucleic acids are stable over a range of temperatures for long-term storage while remaining available for genetic analysis (16). For example, FTA^®^ cards have been used to transport samples of oral fluid, whole blood, sera, and nasal secretions containing the porcine reproductive and respiratory syndrome virus (PRRSV), as well as whole blood for swine influenza and African swine fever (17–21). Additionally, FTA^®^ cards have been implemented in the diagnosis of numerous veterinary bacterial pathogens (21–25), including members of the *Pasteurellaceae* (16, 26), but not (to our knowledge) *A. pleuropneumoniae*. Therefore, in order to reduce biosafety concerns and enable rapid and affordable diagnostics of this important swine pathogen, we have developed and validated an FTA^®^ card protocol for processing clinical samples or bacterial cultures, which can be coupled to our previously described APP-mPCRs (10, 12) for molecular detection and typing of *A. pleuropneumoniae*.

## Materials and Methods

### Bacterial Strains and Growth Conditions

*A. pleuropneumoniae* reference strains for serovars 1–19 (4074, S1536, S1421, M62, K17, L20, Femø, WF83, 405, CVJ13261, D13039, 56153, 1096, N273, 3906, HS143, A-85/14, 16287-1, 7311555, and 7213384-1, respectively), the UK serovar 8 strain MIDG2331, and 85 clinical isolates originating from porcine lungs were cultured from our laboratory stock collection for this study. *A. pleuropneumoniae* isolates were grown at 37°C overnight in a 5% CO_2_ environment on Bacto Brain Heart Infusion (BHI) broth (BD, Berkshire, England, UK, Cat #2237500) containing 15 g/l agar (Sigma-Aldrich, Gillingham, Dorset, UK, Cat #W201201) and supplemented with 0.01% nicotinamide adenine dinucleotide (NAD) (MP Biomedicals, Eschwege, Germany, Cat #100319).

### Lung Sample Collection and *A. pleuropneumoniae* Strain Isolation

Initially, 10 fresh lung samples, from farms (in England or Northern Ireland) with a history of clinical pleuropneumonia, were selected for validation of culture-free detection and typing of *A. pleuropneumoniae* using gDNA extracted directly from the tissue vs. typing of extracted gDNA from bacteria cultured from the tissues. For these, the tissue surface over the lesioned area was disinfected with 70% ethanol prior to making an aseptic incision and the lesion being swabbed. The swabs were subsequently inoculated onto BHI-NAD agar plates that were cultivated in the presence of 5% CO_2_ at 37°C overnight.

Subsequently, to compare the performance of gDNA extracted directly from lung samples to tissue imprints made on FTA^®^ cards, 22 lung samples were collected from pigs with suspected *A. pleuropneumoniae* infection, or during clinical outbreaks of swine pleuropneumonia, from a total of 13 farms located in either England or Portugal. The fresh lung samples were either processed immediately or stored at −20°C and defrosted thoroughly prior to processing for preparation of FTA^®^ card samples and extraction of gDNA (see sections *Evaluation of A. pleuropneumoniae inactivation by FTA*^^®^^ *cards* and *DNA extraction from bacterial culture and lung tissue* below).

### Bacteria and Tissue Sample Application Onto FTA^®^ Cards

For cultured *A. pleuropneumoniae*, bacterial suspensions were prepared by resuspending growth from overnight plate cultures in phosphate-buffered saline (PBS) to OD_600_ = 1.0. For each suspension, 100 μl were pipetted onto the FTA^®^ classic card (Whatman plc, Little Chalfont, Buckinghamshire, UK, Cat #WB120205), ensuring even sample distribution. Initially, FTA^®^ cards inoculated with either the serovar 19 reference strain, 7213384-1, or the serovar 8 strain, MIDG2331, were used for optimization of processing and PCR conditions. Subsequently, FTA^®^ cards were prepared for all 19 serovar reference strains, as well as 85 *A. pleuropneumoniae* isolates cultured from the clinical lung samples, in order to validate detection and typing by APP-mPCR1 and APP-mPCR2, as described below. For the 22 lung tissue samples, following surface decontamination with 70% ethanol, visibly lesioned (where possible) areas were excised from the fresh or defrosted lung tissue using a sterile disposable scalpel (Swann-Morton, Sheffield, South Yorkshire, UK, Cat #0514). A tissue smear was made onto FTA^®^ cards by pressing the sample area of the cards against the surface of the excised tissue (repeatedly, if necessary) until the card appeared visibly saturated. All samples on FTA^®^ cards were dried in a biosafety cabinet (Class A II) for at least 2 h and then stored in resealable plastic bags in a laboratory cupboard until used in the mPCRs.

### Evaluation of *A. pleuropneumoniae* Inactivation by FTA^®^ Cards

To validate inactivation of *A. pleuropneumoniae*, discs punched (see below) from FTA^®^ cards inoculated with various concentrations of the UK serovar 8 strain MIDG2331 (as an exemplar) were placed on the surface of BHI-NAD plates to check for bacterial growth. Briefly, a suspension (OD_600_ = 1.0) of MIDG2331 was prepared by collecting growth from an overnight plate culture, suspended into PBS (Sigma-Aldrich, Cat #p4417). Serial 10-fold dilutions were then made in PBS, with aliquots plated on BHI-NAD to determine the colony-forming units per milliliter, and 100 μl of each concentration (including undiluted) spotted separately onto the sample area of FTA^®^ cards. These cards were allowed to dry completely in a Class A II biosafety cabinet at room temperature for 2 h. Triplicate 3-mm discs were obtained from each card using a sterile biopsy punch (Integra Miltex Cat #12-460-406, Fisher Scientific, Loughborough, UK) and transferred onto BHI-NAD plates. The plates were kept in an incubator with 5% CO_2_ at 37°C for 1 week and checked daily for bacterial growth. Inactivation was also confirmed with FTA^®^ cards inoculated with two different *A. pleuropneumoniae*-positive lung smears.

### DNA Extraction From Bacterial Culture and Lung Tissue

Approximately 50 mg of 10 additional lung tissues (containing visible lesions where possible) were collected with a disposable sterile scalpel and homogenized in a lysing matrix A 2-ml microcentrifuge tube (MP Biomedicals, Cat #116910050-CF) with 360 μl ATL buffer (Qiagen Ltd., Manchester, UK, Cat #19076) at a setting of 6.0 m/s for 60 s using a FastPrep-24 5G (MP Biomedicals, Cat #116005500). Subsequent extraction of DNA was then performed using a QIAamp DNA Mini Kit (Qiagen Ltd., Cat #51304), following the manufacturer's instructions. For isolated *A. pleuropneumoniae* cultures, half of a 10-μl loop of bacterial colonies was collected from overnight BHI-NAD plate cultures, and DNA extraction was performed using a QIAamp DNA Mini Kit.

### Preparation of FTA^®^ Discs for PCR Amplification

Discs from each FTA^®^ card, inoculated with either lung smears or bacterial cultures, were punched using a 3-mm biopsy punch (Integra Miltex). The biopsy punch was cleaned between each use by rinsing in distilled water and 70% ethanol to minimize cross contamination between samples. Each disc was subsequently transferred to individual 1.5-ml microtubes for evaluation of different wash conditions prior to use in PCR. We compared the FTA^®^-recommended 30-min wash protocol whereby discs were washed three times in FTA^®^ purification reagent (Whatman plc, Cat #WHAWB120204), then twice in TE buffer (10 mM Tris, 1 mM EDTA, pH 8.0); after which, they were dried by heating at 55°C for 15 min (16, 27), with the use of only distilled water (evaluated for number, volume, and duration of washes) with no drying. Subsequently, our optimized water-wash protocol, where the discs were subjected to two (for bacterial cultures), or three (for tissue smears), 5-min washes in 1 ml of distilled water, prior to immediate transfer to PCR tubes, was used for further evaluation of FTA^®^ cards for *A. pleuropneumoniae* diagnostics with our mPCRs.

### FTA^®^ Card-mPCR Sensitivity

Limit of detection was initially evaluated using FTA^®^ cards inoculated with different concentrations of cultured bacteria. Briefly, colonies from an overnight plate culture of serovar 8 strain MIDG2331 were washed in PBS and resuspended to an OD_600_ = 1.0. Seven 10-fold serial dilutions were made. Aliquots of each dilution were plated on BHI-NAD to determine the colony-forming units per milliliter of *A. pleuropneumoniae*, and 100 μl of each (including undiluted) suspension were added to FTA^®^ cards and allowed to dry fully prior to processing. Following washing (comparing our optimized water-wash protocol to the FTA^®^-recommended protocol, as described above), one disc from each of the serial dilution samples was transferred to a 50-μl PCR reaction for direct amplification by mPCR.

Additionally, homogenized tissue samples (15 mg/ml in PBS) from a control lung (negative by APP-mPCR1 and APP-mPCR2), spiked with different concentrations of cultured MIDG2331 suspensions prepared above, were used to determine the limit of detection of *A. pleuropneumoniae* following gDNA extraction of the tissue suspensions, as well as following their application to FTA^®^ cards. For preparation of the spiked homogenate samples, 100 μl of each dilution of the MIDG2331 suspension were inoculated into respective 1-ml aliquots of lung homogenate, to give a further 1/10 dilution. For each inoculated homogenate suspension, 100 μl were applied to FTA^®^ cards, and 100 μl were processed for DNA extraction, i.e., homogenized with 360 μl ATL buffer (Qiagen Ltd.) in a lysing matrix A 2-ml microcentrifuge tube (MP Biomedicals), and vortexed thoroughly for 2 min prior to the use of the QIAamp DNA Mini Kit (Qiagen Ltd.), as described above. The inoculated FTA^®^ cards were dried and processed (again comparing the two different wash protocols) for use in APP-mPCRs.

### *A. pleuropneumoniae* PCR Amplification

For optimization of FTA^®^ card processing conditions, single-plex PCR reactions were prepared with either a PCR kit containing HotStarTaq DNA Polymerase (Qiagen Ltd., Cat #206152) or DreamTaq HotStart DNA Polymerase (Thermo Fisher UK, Loughborough, UK, Cat #K9011) in 50 μl total reaction volumes (prepared according to manufacturers' protocols), with serovar 19-specific primers (12) at a final concentration of 0.2 μM of each, and one prepared 3-mm sample disc per tube. Multiplex PCRs for *A. pleuropneumoniae* identification and typing were performed with the previously published APP-mPCR1 and APP-mPCR2 primer sets (12), using the Qiagen Multiplex Plus PCR Kit (Qiagen Ltd., Cat #206152), in 50 μl total reaction volumes consisting of 25 μl 2X Master Mix (containing HotStarTaq DNA Polymerase), 0.2 μM of each primer, 5 μl 10x CoralLoad gel-tracking dye (Qiagen Ltd.), and either one prepared sample disc or 4 μl gDNA. Reactions were run on a T100 Thermal Cycler (Bio-Rad, Cressier, Switzerland, Cat #1861096) for 10 min at 95°C, followed by 30 cycles of 95°C for 15 s, 60°C for 90 s, and 72°C for 150 s, with a final extension at 68°C for 10 min. After amplification, 10-μl aliquots of the PCR products were separated by gel electrophoresis using 1.5% (w/v) agarose gels. A GeneRuler 100 bp Plus DNA Ladder (Thermo Fisher UK, Cat #SM0321) was included for band size determination of PCR products. The gels were stained with ethidium bromide, visualized under ultraviolet light, and photographed using a Bio-Rad Gel Imaging System.

## Results

### Optimization of an FTA^®^ Card-PCR Protocol for *A. pleuropneumoniae* Diagnostics

Rapid and complete inactivation of *A. pleuropneumoniae* viability by the FTA^®^ card surface chemistry was confirmed, as no bacterial growth was observed for discs from cards inoculated with up to 5 × 10^9^ CFU/ml of strain MIDG2331 and allowed to dry for 2 h prior to plating on BHI-NAD. Furthermore, no bacterial colonies were observed for discs from FTA^®^ cards inoculated with *A. pleuropneumoniae*-positive lung smears.

**Figure 1:**
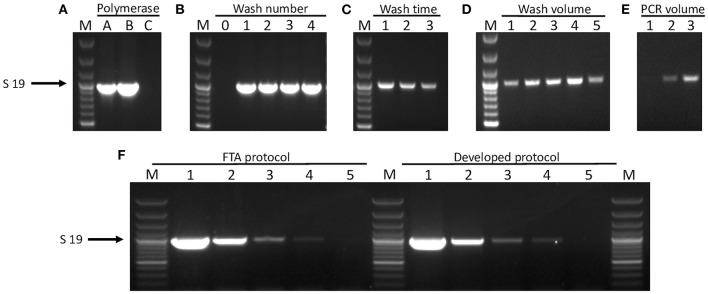
Optimization of FTA^®^ card processing for direct PCR amplification. FTA^®^ card discs inoculated with reference strain 7213384-1 at OD_600_ = 0.01 producing serovar 19-specific product are shown for different optimization conditions. **(A)** PCR amplification from FTA^®^ cards using different polymerases: A = HotStarTaq^^®^^Plus Polymerase, B = HotStarTaq^^®^^ Polymerase, and C = DreamTaq Polymerase. **(B)** The number of washes in “deionized water” required to achieve amplification: 0–4 = no wash to 4 washes, respectively. **(C)** Length of incubation with deionized water per wash: 1 = 5 min, 2 = 10 min, and 3 = 15 min. **(D)** Altering the volume of water used to wash in a single step for 5 min: 1 = 100 μl, 2 = 200 μl, 3 = 300 μl, 4 = 400 μl, and 5 = 500 μl. **(E)** Various PCR reaction volumes: 1 = 30 μl, 2 = 40 μl, and 3 = 50 μl. **(F)** Limit of detection is shown for amplification of the serovar 19-specific product from discs inoculated with strain 7213384-1. Lanes labelled 1–5 are for OD_600_ = 0.01 and four subsequent 10-fold dilutions, respectively, of bacterial culture on discs prepared either with the Whatman FTA^®^ protocol (left hand lanes) or our developed wash protocol (right hand lanes). For all gels, lane M = 100 bp Plus DNA Ladder (GeneRuler, Thermo Fisher Scientific).

Factors affecting amplification of *A. pleuropneumoniae* target genes from DNA captured on FTA^®^ cards included choice of polymerase and PCR reaction volume, as well as the number, volume, and duration of washes in distilled water (Figures 1A–E). Using the Qiagen Multiplex PCR Kit mix (containing HotStarTaq) in 50-μl reaction volumes, discs from cards inoculated with bacterial culture and processed with our optimized wash protocol (i.e., two 5-min washes in 1 ml of distilled water, with undried discs immediately used in PCR) achieved a limit of detection of 1.64 × 10^3^ CFU per 3 mm FTA^®^ card disc, equating to an OD_600_ of 0.0001, or 7.3 × 10^4^ CFU/ml (Figure 1F). A similar sensitivity was achieved with the 30-min FTA^®^-recommended protocol of three washes in FTA^®^ purification buffer and two washes in TE buffer, with drying of discs at 55°C for 15 min prior to PCR (Figure 1B). For *A. pleuropneumoniae* cultures spiked into homogenized lung tissue, we achieved a limit of detection of 3.83 × 10^3^ CFU per 3 mm FTA^®^ card disc equating to 1.7 × 10^5^ CFU/ml. In a comparison of APP-mPCR detection using discs from FTA^®^ cards inoculated with dilutions of cultured bacterial in lung homogenate, with the same suspensions extracted with a commercial kit, a decrease (100-fold) in detection was seen (Supplementary Figure 1). However, a comparison of amplification from FTA^®^ cards inoculated with the suspensions in lung homogenate vs. suspensions made in PBS only indicated a 10-fold decrease (beyond that introduced by inoculation of the suspension into the homogenate) in detection (data not shown). This suggested that the FTA^®^ card itself may be affecting the sensitivity, and an additional (i.e., third) 5-min wash in 1 ml of distilled water was found to be optimal for processing lung smear-inoculated FTA^®^ cards vs. those inoculated with bacterial culture suspensions.

The stability of *A. pleuropneumoniae* DNA captured on FTA^®^ cards was confirmed for both cultured bacterial samples and lung smears, with amplification of target sequences being detectable after 24 weeks at 37°C, or 2 years at room temperature (data not shown). Using clinical samples (four lung smears), the same APP-mPCR result (i.e., one each of serovars 4 and 5 and two negative samples) was achieved for purified gDNA as for matched samples on FTA^®^ cards, even after 2 years of storage at room temperature. However, the level of amplification was decreased (with regard to band intensity) for FTA^®^ cards stored at room temperature for over a year (data not shown).

### Validation of FTA^®^ Card Samples for Use in APP-mPCR1 and APP-mPCR2

Of the 10 lung samples used to validate culture-free detection and typing of *A. pleuropneumoniae* from clinical lung tissue, two yielded no growth, four indicated pure cultures typical of *A. pleuropneumoniae*, and four resulted in mixed bacterial cultures when plated on BHI-NAD. Subsequent serotyping by APP-mPCR indicated serovar 5 for one of the pure cultures, whereas serovar 8 was detected for the other three pure as well as the four mixed bacterial cultures. In all cases, the results matched those obtained using extracted gDNA from the same lung samples. Additionally, serovar 8 was detected in the gDNA from the two lung tissue samples for which no bacterial growth was obtained, indicating that culture-free detection was more sensitive than bacteriological isolation.

**Figure 2:**
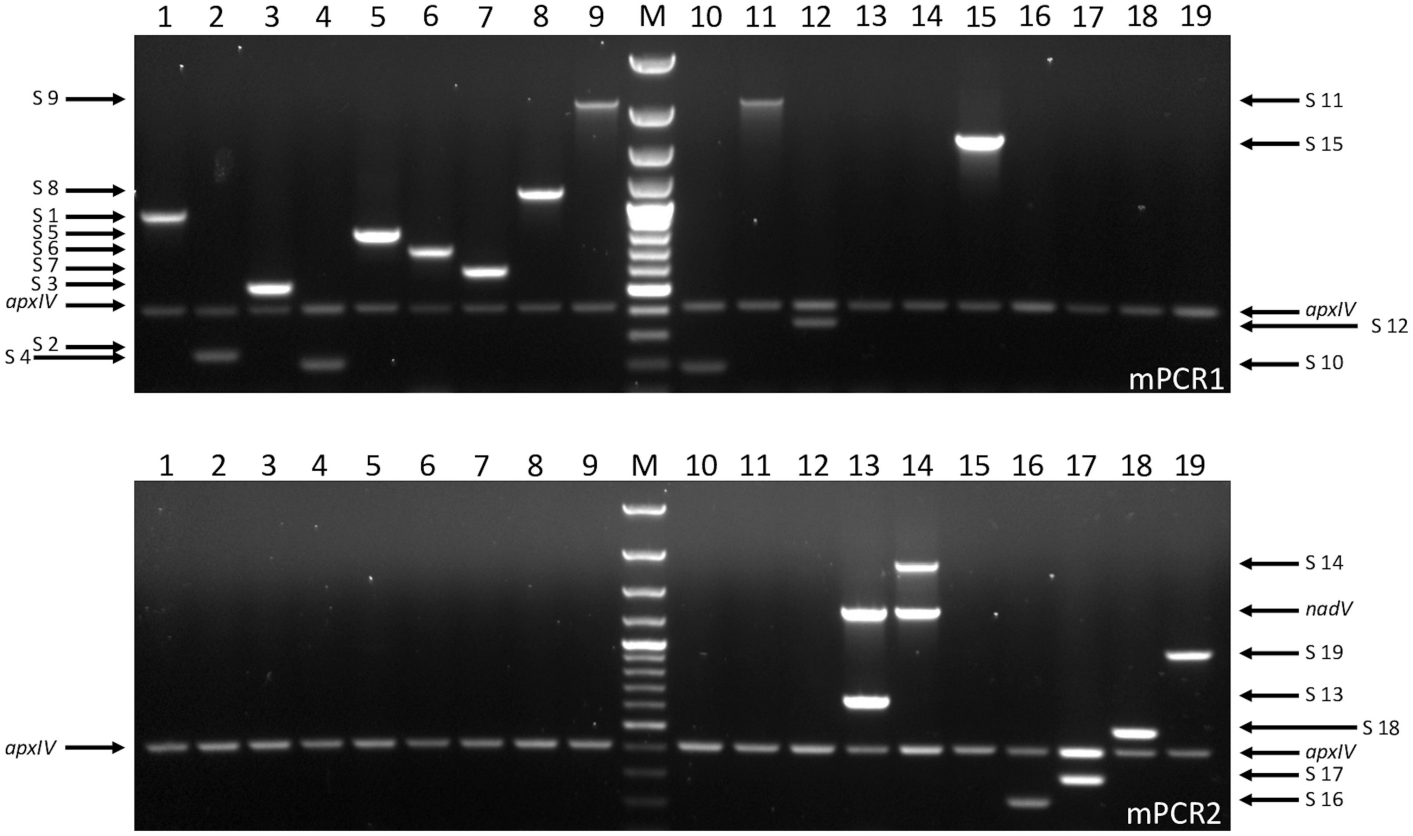
Serovar-specific products amplified by APP-mPCR1 (upper) and APP-mPCR2 (lower) using samples from FTA^®^ cards inoculated with *A. pleuropneumoniae* reference strains for serovars 1–19 (1, 4074T; 2, S1536; 3, S1421; 4, M62; 5a, K17; 5b, L20; 6, Femø; 7, WF83; 8, 405; 9, CVJ13261; 10, D13039; 11, 56153; 12, 8329; 13, N-273; 14, 3906; 15, HS143; 16, A-85/14; 17, 16287-1; 18, 7311555; and 19, 7213384-1) at OD_600_ = 0.01. An *apxIV* (423-bp) amplicon is detected in all 19 serovar reference strains, with an additional 1,339-bp *nadV* amplicon detected only in the biovar 2 isolates (lanes 13 and 14). For both gels, lane M = 100 bp Plus DNA Ladder (GeneRuler, Thermo Fisher Scientific).

**Figure 3:**
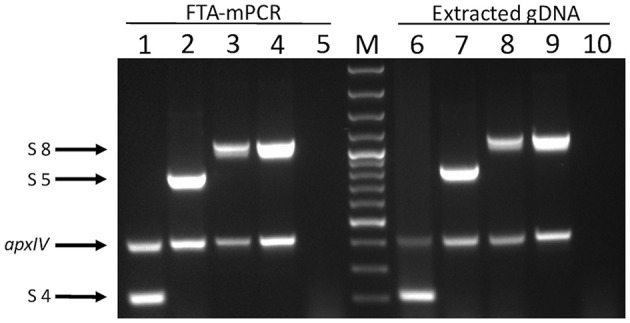
Products of APP-mPCR1 amplified directly from lung tissue imprints on FTA^®^ cards (lanes 1–5) and extracted genomic material extracted using commercial kits from the same lung tissues (lanes 9–10). Lanes 1 and 6 = serovar 4; lanes 2 and 7 = serovar 5; lanes 3, 4, 7, and 9 = serovar 8; and lanes 5 and 10 = an APP-negative lung. Lane M = 100 bp Plus DNA Ladder (GeneRuler, Thermo Fisher Scientific).

In order to validate the use of FTA^®^ cards for simpler, faster, culture-free detection and typing of *A. pleuropneumoniae* from clinical lung samples, we initially confirmed that all 19 *A. pleuropneumoniae* serovar reference strains could be successfully typed by our APP-mPCRs (Figure 2) using discs from FTA^®^ cards inoculated with bacterial culture suspensions. Furthermore, a comparison of APP-mPCR results obtained for gDNA extracted from 85 clinical isolates using the QIAamp DNA Mini Kit to results of APP-mPCRs performed using inoculated FTA^®^ cards showed 100% correlation (Supplementary Table 1). Finally, the APP-mPCR results for 22 clinical lung tissue samples extracted using the QIAamp DNA Mini Kit showed 100% correlation with results obtained using FTA^®^ cards inoculated with matched lung smears (Table 1). Of the 22 lung samples from animals with suspected *A. pleuropneumoniae* infection, 15 produced amplicons for the species-specific *apxIV* gene for both gDNA and matched lung smear-inoculated FTA^®^ cards, confirming the presence of *A. pleuropneumoniae* (Figure 3). Of these positive samples, the same serovar-specific amplicons were also detected for both gDNA and FTA^®^ card discs, with serovar 8 detected for samples from England and serovars 4, 5, and 17 detected for samples from Portugal (Table 1).

**Table 1:** Clinical samples with suspected *A. pleuropneumoniae* infection.

**Sample**	**Number of samples (*n*)**	**Geographicalorigin**	**gDNA-mPCR**	**FTA^^®^^-mPCR**
			**Amplicon**	**Amplicon**
Farm 1	1	Portugal	Serovar 5	Serovar 5
Farm 2	1	Portugal	Serovar 4	Serovar 4
Farm 3	1	Portugal	Serovar 5	Serovar 5
Farm 4	3	England	APP negative	APP negative
Farm 5	3	England	Serovar 8	Serovar 8
Farm 6	3	England	APP negative	APP negative
Farm 7	1	Portugal	Serovar 5	Serovar 5
Farm 8	1	England	APP negative	APP negative
Farm 9	2	England	Serovar 8	Serovar 8
Farm 10	1	England	Serovar 8	APP negative
Farm 11	1	England	Serovar 8	APP negative
Farm 12	3	Portugal	Serovar 17	Serovar 17
Farm 13	1	Portugal	Serovar 17	Serovar 17

*Twenty-two lung tissue samples from 13 different farms were obtained for use in this study. APP-mPCR amplification results are shown for both extracted gDNA using a commercial kit and preparation of bacterial isolates or lungs made on FTA^®^ cards*.

## Discussion

With the increasing shift away from serological typing of *A. pleuropneumoniae* isolates (28), which can be problematic due to cross-reactivity caused by shared lipopolysaccharide O-Ags among some groups of serovars (e.g., 1/9/11, 3/6/8/15, and 4/7), our group has been at the forefront of development, validation, and application of *cps* gene-specific mPCRs (10, 12, 29, 30) for detection and typing of all known serovars (currently 19) of this important swine respiratory pathogen. Our APP-mPCR1 and APP-mPCR2 assays have been extensively validated for sensitivity and specificity using purified gDNA, not only from all 19 serovar reference strains but also from a large collection of clinical *A. pleuropneumoniae* isolates from numerous countries, as well as negative control bacterial species commonly found in pigs. Although validated with gDNA from cultured bacteria, we have also been using these mPCRs for diagnostics with gDNA extracted from clinical lung tissue samples, for culture-free detection and typing of *A. pleuropneumoniae*.

Elimination of the requirement for bacterial culture by extracting gDNA directly from infected tissue not only saves time but also increases detection. It is not always possible to isolate *A. pleuropneumoniae* from clinical samples, especially if faster growing bacteria are present (either as co-infections or contaminants acquired during collection and transportation of the sample). Furthermore, in cases where antimicrobial treatment had been administered, bacteria in the lung may be non-viable but can nevertheless still be detected molecularly, making culture-free detection more sensitive in these cases. Yet, even processing tissue samples for gDNA extraction can be laborious and costly, requiring expensive equipment for thorough tissue homogenization and commercial kits for DNA extraction, which may be impractical for resource-limited labs. Additionally, transportation of clinical tissue samples to diagnostic labs is highly regulated, especially when shipping between countries, and requires use of specialized couriers who can deliver samples on either wet or dry ice (needed to reduce overgrowth of contaminant bacteria).

The use of Whatman FTA^®^ cards for collecting and preserving DNA has become a popular means to transport clinical samples to labs for molecular diagnostics. With inactivation of pathogenic organisms *via* a specialized chemical coating, samples on FTA^®^ cards can safely be sent using normal postal services, even internationally, thereby greatly reducing the cost of shipping. Diagnostic samples applied to FTA^®^ cards include cultured microorganisms and infected bodily fluids (e.g., blood, oral fluids, and mucosal secretions). Their use has been validated for a variety of viral and bacterial pathogens (24, 31, 32), and recently, *Escherichia coli, Staphylococcus aureus*, and *Pasteurella multocida* have been identified from impression smears of internal organs from chickens collected on FTA^®^ cards (33).

To our knowledge, this is the first study to describe the use of FTA^®^ cards for collecting and mPCR-serotyping of *A. pleuropneumoniae* samples directly from clinical lung tissues. We have confirmed that FTA^®^ cards effectively inactivate *A. pleuropneumoniae* from purified bacterial cultures as well as clinical lung samples. Although not examined in our current study, the detergent coating of FTA^®^ cards has previously been shown to lyse viruses including PRRSV, swine influenza, and African swine fever (17–21), therefore abrogating the potential risk when transporting clinical samples derived from pigs that may have viral co-infection, common in cases of the porcine respiratory disease complex (34), especially in countries suffering from high rates of endemic disease.

In this investigation, we have validated the performance of our previously described APP-mPCRs with a new, substantially simplified, protocol for processing and testing of FTA^®^ cards, which will allow the safe collection, transportation, and rapid serotyping of *A. pleuropneumoniae* from clinical samples, or cultured bacterial isolates, without DNA extraction. Prior to use in PCR, discs punched from inoculated FTA^®^ cards are typically washed with a proprietary FTA^®^ purification reagent (Whatman), followed by Tris-EDTA, in a process taking 30 min (16, 27). In contrast, we have found that two brief (5-min) washes with distilled water provided consistent results for the detection of *A. pleuropneumoniae* DNA from FTA^®^ cards inoculated with pure cultures, with a third wash added for optimal results when using cards inoculated with lung smears. This wash protocol was optimized for resource-limited settings, minimizing chemical usage and overall time required for the wash steps compared to the Whatman FTA^®^ purification protocol. Our wash protocol significantly reduced the disc processing time, including elimination of the recommended 15-min drying step at 55°C, with no difference in amplification found when transferring washed discs to PCR reactions directly after washing or after waiting for the discs to air dry at room temperature.

Our protocol was found to yield sensitive and reproducible amplification of the *A. pleuropneumoniae* diagnostic *apxIV* and serovar-specific *cps* gene products (with sizes ranging from 183 to 2,105 bp) from cultured bacterial samples applied to FTA^®^ cards. A slight decrease in sensitivity was seen for detection of *A. pleuropneumoniae* in spiked lung homogenate samples compared to suspensions in PBS, suggesting the possible presence of trace PCR inhibitors in the tissue samples. However, a comparison of results for detection and typing of *A. pleuropneumoniae* from clinical samples showed 100% correlation between template gDNA prepared from homogenized lung tissue and matched sample discs from lung smear-inoculated FTA^®^ cards, indicating that if any PCR inhibitors were occasionally present in the tissue samples on the FTA^®^ cards, they were sufficiently removed by washing so as to not interfere with amplification of the targets present.

Using gDNA from clinical tissue, we demonstrated that culture-free PCR results were in agreement with those for amplification using gDNA extracted from recovered pure and mixed bacterial cultures for matched lung samples and additionally detected serovar 8 in two samples from which no bacteria were recovered, indicating greater sensitivity. Of the 22 clinical lung samples from animals with suspected *A. pleuropneumoniae* infection, 15 were positive in our mPCRs and the remaining seven were negative, with no detection even of the species-specific *apxIV* gene, indicating that either a different pathogen was responsible for the clinical disease or the level of *A. pleuropneumoniae* present was below the level of detection. It may be possible to achieve greater sensitivity of detection of *A. pleuropneumoniae* using quantitative PCR (qPCR) rather than mPCR. However, for use in qPCR, it would be necessary to extract the DNA from FTA cards, rather than use a punched disc directly, as we have done with our mPCR assays. Furthermore, to date, diagnostic qPCRs for *A. pleuropneumoniae* have only been described for detection of the species-specific *apxIV* gene (35) and serotyping of a limited number of serovars (36, 37). Further work will be required to extend diagnostic qPCRs to allow comprehensive serotyping of *A. pleuropneumoniae* compatible with the use of FTA card samples. With our set of two validated mPCRs, we were able to identify that the seven samples collected from four farms in England were all serovar 8, whereas serovars 4, 5, and 17 were detected from one, three, and four samples, respectively, originating from Portugal. The serovar 5 isolates were from three separate Portuguese farms, and the serovar 17 isolates were from two others. Where multiple isolates were obtained from the same farm, they were of the same serovar in each case. Use of discs from FTA^®^ cards inoculated with lung smears gave the same results as gDNA extracted from homogenized tissue, validating the use of FTA^®^ cards for collection of samples directly from infected tissue without the need for culture or gDNA extraction. Furthermore, the culture-free PCR results were in agreement with those for amplification from recovered pure and mixed bacterial cultures for matched lung samples and additionally detected serovar 8 in two samples from which no bacteria were recovered, indicating greater sensitivity. *A. pleuropneumoniae*-containing samples on FTA^®^ cards were shown to be stable for long periods of time over a range of temperatures, including 24 weeks at 37°C, which will facilitate their implementation in warmer climates, such as the Philippines, where a recent increase in high-intensity pig farming has resulted in a large increase in respiratory infection outbreaks (38).

In conclusion, we have established a simplified water-based method for processing FTA^®^ cards, inoculated either with bacterial culture or by impression with infected tissue, for direct detection and typing of *A. pleuropneumoniae* serovars 1–19 in our recently improved APP-mPCR1 and APP-mPCR2 assays (12). The use of FTA^®^ cards allows *A. pleuropneumoniae* samples to be transported by non-specialized national and international postal services, providing an economical alternative to transporting these biohazardous samples. By developing this simple, cost-efficient, and sensitive detection approach, we hope to increase *A. pleuropneumoniae* diagnostic efforts, facilitating epidemiological studies and efficacious vaccination programs to combat this economically important disease. Furthermore, the protocol developed here may have broader applications to other pathogenic microorganisms, especially those of the porcine respiratory disease complex.

## Data Availability Statement

The original contributions presented in the study are included in the article/Supplementary Material, further inquiries can be directed to the corresponding author/s.

## Author Contributions

All authors made significant contribution to the study concept. OS and YL designed and performed experiments including data analysis. OS, JB, YL, and PL contributed to writing the manuscript. SL, MG, LF, ØA, EV, PP, and LL provided bacteria strains or clinical samples and were involved in proofreading the manuscript. PL secured funding. All authors contributed to the article and approved the submitted version.

## Conflict of Interest

EV and PP are employed by Ceva Animal Health Ltd. The remaining authors declare that the research was conducted in the absence of any commercial or financial relationships that could be construed as a potential conflict of interest.

## Publisher's Note

All claims expressed in this article are solely those of the authors and do not necessarily represent those of their affiliated organizations, or those of the publisher, the editors and the reviewers. Any product that may be evaluated in this article, or claim that may be made by its manufacturer, is not guaranteed or endorsed by the publisher.
