# Supplementary material for: Rapid Detection and Typing of Actinobacillus pleuropneumoniae Serovars Directly From Clinical Samples: Combining FTA® Card Technology With Multiplex PCR
Source: Front Vet Sci. 2021 Aug 10;8:728660. doi: 10.3389/fvets.2021.728660 (PMC8382971; doi:10.3389/fvets.2021.728660)
Supplement: Supplementary file 1 [file Table_1.DOCX]

Supplementary Material

**Supplementary Figure 1.** Products of APP-mPCR1 amplified from lung homogenate spiked with *A. pleuropneumoniae* inoculated onto an FTA® card compared to DNA-extracted using a commercial kit. Lanes 1-7 and 8-14 show PCR product from 10-fold serial dilutions starting from OD_600_ 1.0 of serovar 8 isolate, MIDG2331, made in PBS and spiked into homogenized lung tissue. Lanes 1-7 show amplified extracted gDNA from the spiked lung homogenate. Lanes 8-14 show amplification from 3 mm punches of FTA® cards inoculated with the same spiked homogenate. Lane M = 100 bp Plus DNA ladder (GeneRuler, Thermo Fisher Scientific).

**Supplementary Table 1. APP-mPCR serotyping of *A. pleuropnuemoinae* clinical isolates.** APP-mPCR results from 85 *A. pleuropneumoniae* clinical isolates obtained from our culture collection, with amplification from FTA® card inoculated with culture or using extracted gDNA.

| **Serotype** | **Amplified using FTA® Card (n)** | **Amplified using bacterial DNA (n)** |
| --- | --- | --- |
| **1** | **2** | **2** |
| **2** | **13** | **13** |
| **3** | **1** | **1** |
| **4** | **16** | **16** |
| **5** | **4** | **4** |
| **6** | **3** | **3** |
| **7** | **2** | **2** |
| **8** | **1** | **1** |
| **9/11** | **10** | **10** |
| **10** | **1** | **1** |
| **12** | **1** | **1** |
| **13** | **12** | **12** |
| **14** | **1** | **1** |
| **15** | **1** | **1** |

| **16** | **2** | **2** |
| --- | --- | --- |
| **17** | **11** | **11** |
| **18** | **2** | **2** |
| **19** | **2** | **2** |
